# Synthesizing evidence for the external cycling of NO_x_ in high- to low-NO_x_ atmospheres

**DOI:** 10.1038/s41467-023-43866-z

**Published:** 2023-12-02

**Authors:** Chunxiang Ye, Xianliang Zhou, Yingjie Zhang, Youfeng Wang, Jianshu Wang, Chong Zhang, Robert Woodward-Massey, Christopher Cantrell, Roy L. Mauldin, Teresa Campos, Rebecca S. Hornbrook, John Ortega, Eric C. Apel, Julie Haggerty, Samuel Hall, Kirk Ullmann, Andrew Weinheimer, Jochen Stutz, Thomas Karl, James N. Smith, Alex Guenther, Shaojie Song

**Affiliations:** 1 State Key Joint Laboratory of Environmental Simulation and Pollution Control (SKL-ESPC), College of Environmental Sciences and Engineering, Peking University, Beijing, China; 2 Wadsworth Center, New York State Department of Health, Albany, NY USA; 3 Department of Environmental Health Sciences, State University of New York, Albany, NY USA; 4 School of Ecology and Nature Conservation, Beijing Forestry University, Beijing, China; 5 Department of Chemistry, University of Leeds, Leeds, UK; 6 Université Paris-est Créteil, LISA (Laboratoire Interuniversitaire des Systèmes Atmosphériques), Paris, France; 7 Center for Atmospheric Particle Studies, Carnegie Mellon University, Pittsburgh, PA USA; 8 Department of Chemistry, Carnegie Mellon University, Pittsburgh, PA USA; 9 Department of Atmospheric and Oceanic Sciences, University of Colorado Boulder, Boulder, CO USA; 10 National Center for Atmospheric Research, Boulder, CO USA; 11 Department of Atmospheric and Oceanic Sciences, University of California, Los Angeles, CA USA; 12 Institute for Meteorology and Geophysics, University of Innsbruck, Innsbruck, Austria; 13 Earth System Science, University of California, Irvine, CA USA; 14 State Environmental Protection Key Laboratory of Urban Ambient Air Particulate Matter Pollution Prevention and Control & Tianjin Key Laboratory of Urban Transport Emission Research, College of Environmental Science and Engineering, Nankai University, Tianjin, China

**Keywords:** Atmospheric chemistry, Environmental impact

## Abstract

External cycling regenerating nitrogen oxides (NO_x_ ≡ NO + NO_2_) from their oxidative reservoir, NO_z_, is proposed to reshape the temporal–spatial distribution of NO_x_ and consequently hydroxyl radical (OH), the most important oxidant in the atmosphere. Here we verify the in situ external cycling of NO_x_ in various environments with nitrous acid (HONO) as an intermediate based on synthesized field evidence collected onboard aircraft platform at daytime. External cycling helps to reconcile stubborn underestimation on observed ratios of HONO/NO_2_ and NO_2_/NO_z_ by current chemical model schemes and rationalize atypical diurnal concentration profiles of HONO and NO_2_ lacking noontime valleys specially observed in low-NO_x_ atmospheres. Perturbation on the budget of HONO and NO_x_ by external cycling is also found to increase as NO_x_ concentration decreases. Consequently, model underestimation of OH observations by up to 41% in low NO_x_ atmospheres is attributed to the omission of external cycling in models.

## Abstract (web summary)

External cycling regenerates nitrogen oxides from the NO_x_ oxidative reservoir, NO_z_. Aircraft observations reveal NO_x_ external cycling compensates for NO_x_ aging, sustaining NO_x_ distribution and production of OH radicals far from NO_x_ emission sources

## Introduction

Nitrogen oxides (NO_x_ ≡ NO + NO_2_) and gaseous nitrous acid (HONO) perturb the photochemical cycling of peroxy radicals (RO_2_ and HO_2_) and hydroxide radicals (OH)^1–6^. Field observations in high-NO_x_ atmospheres highlight the primary production of OH (and NO) via HONO photolysis^1,6–10^. Across high- to low-NO_x_ atmospheres, secondary production of OH via the HO_2_ plus NO reaction is another major source of OH.

**Fig. 1:**
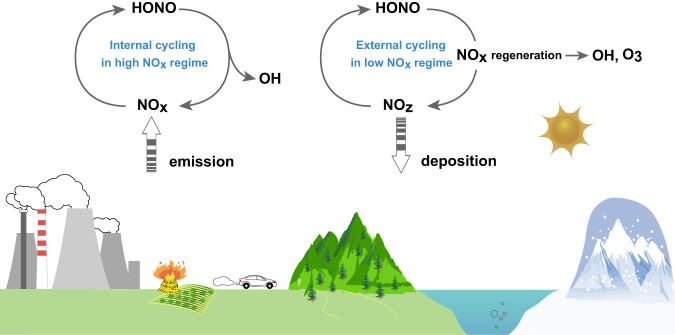
Schematic graph of internal cycling and external cycling of NO and HONO. The left reaction scheme describes internal cycling between nitrogen oxides (NO = NO + NO) and nitrous acid (HONO) in high- NO atmospheres. HONO photolysis is the major route producing OH radical (OH). The right reaction scheme describes external cycling among oxidative reservior species of NO (NO), NO, and HONO in low-NO atmospheres. NO -catalysed radical chain propagration takes over as the major route perturbing OH and ozone (O) chemical production.

HONO and NO_x_ are closely coupled in their NO_x_-HONO internal cycling, referred to as internal cycling in this context (Fig. 1). Specifically, heterogeneous reactions of NO_2_ on ambient surfaces and gas-phase reactions between NO and OH have been intensively examined as major HONO formation routes in the internal cycle^7–15^. Since HONO photolyzes much faster, turnover routes of NO_x_ to recycle HONO are the rate-limiting steps of internal cycling. OH is a net product in internal cycling even if only HONO production via heterogeneous reactions of NO_2_ is considered. Any external source (or sink) of HONO or NO_x_ would promote (or suppress) the internal cycling. Apart from the internal cycling, NO_x_ ages to form more oxidized reservoirs, which are referred to as NO_z_, as air masses are transported away from source regions. NO_x_ aging processes suppress the internal cycling and diminish the role of HONO photolysis in the OH budget. However, model underestimation of the NO_x_/NO_z_ ratio observations suggests unknown or underappreciated NO_x_ regeneration pathways from NO_z_ in low-NO_x_ atmospheres^4,16–28^. NO_x_ regeneration pathways, in contrast to the internal cycling, produce “new” NO_x_ and hence are designated as the external cycling of NO_x_ (Fig. 1). External cycling naturally promotes the internal cycling via at least an external source of NO_x_. Consequently, secondary OH production via NO_x_ regeneration and net primary OH production via HONO regeneration could greatly perturb the OH photochemical budget. The current model omission of external cycling would lead to an underestimation of OH production and OH abundance, especially in low-NO_x_ atmospheres.

Identifying and exploring a specific mechanism is a natural step in characterizing external cycling and quantifying its impact on the oxidative capacity of the atmosphere. The external cycling pathways proposed in the literature include at least three mechanisms, i.e., surface-catalyzed photolysis of absorbed nitrate (nitrate_abs_) on snow/ice surfaces and possibly also on aerosol/ambient surfaces^4,20,24,27–38^, nitrification/denitrification in the soil^16,19^ and the thermal decomposition of peroxyacetyl nitrate (PAN). Among these mechanisms, surface-catalyzed photolysis of nitrate_abs_ on aerosol surfaces, referred to as pNO_3_ photolysis, occurs in situ in the air column and therefore potentially perturbs the distribution of NO_x_ and hence the oxidative capacity of the atmosphere from the lower troposphere to the upper troposphere^35^. pNO_3_ photolysis has been intensively explored in laboratory and field studies^4,18,21–23,34,36,37,39^. The reaction rate constant of pNO_3_ photolysis varies over at least two orders of magnitude in high-to low-NO_x_ atmospheres by employing a budget analysis for either HONO or NO_2_ and by assuming that pNO_3_ photolysis fully accounts for the missing source of HONO or NO_2_ in the field^4,21,32,34,38^. Laboratory studies on a variety of pNO_3_ samples have confirmed that pNO_3_ photolysis is greatly enhanced compared to that of gaseous HNO_3_ and that the pNO_3_ photolysis rate constant is highly variable, over 3 orders of magnitude^30,36,37^ Based on these laboratory and field studies, Ye et al. and Andersen et al. have also revealed that pNO_3_ photolysis is surface-catalyzed in nature and is greatly affected by the physicochemical properties of aerosol particles, such as pNO_3_ loading, chemical composition and particle size^30,38^. Efforts in characterizing atmospheric aerosol properties and their photochemical reactivities are critical in quantitatively understanding the role of pNO_3_ photolysis in external cycling. However, the limited availability of the pNO_3_ photolysis rate constant in only a few atmospheric environments, its large variability, and potentially large uncertainties make it difficult to extrapolate results from laboratory studies to field studies or directly compare results among field studies^18,21,22,30,33,34,36–39^. The variability and potentially large uncertainties in the rate constant have also precluded as yet confident confirmation of pNO_3_ photolysis or other reactions as the dominant mechanism of the external cycling or direct characterization of the external cycling in the atmosphere. To solve this dilemma, we suggest synthesizing critical observational and model evidence, summarizing the fundamental characteristics, and quantifying the impact of the external cycling on the oxidative capacity of the atmosphere in high- to low-NO_x_ atmospheres through a broader lens rather than attempting to establish the kinetics or the dominant mechanism of the external cycling.

## Results and discussion

### Synthesized field evidence for external cycling

A comprehensive dataset obtained from an aircraft measurement campaign provided excellent insight into the external cycling of NO_x_ and its perturbation on the oxidative capacity of the atmosphere^4,32^. Nineteen research flights were conducted onboard NSF/NCAR C-130 to collect measurements of NO_x_, HONO, nitric acid (HNO_3_), particulate nitrate (pNO_3_), some alkyl nitrates, PAN, radicals (OH, HO_2_, RO_2_), ozone (O_3_), volatile organic compounds (VOCs), aerosol size distributions, photolysis frequencies, and other meteorological parameters, mostly in various locations in the low-NO_x_ troposphere (Methods), i.e., from the pristine terrestrial and marine boundary layer (BL) to the FT (Fig. S1 and Table S1). Herein, we defined low-NO_x_ and high-NO_x_ regimes with a NO_x_ concentration threshold of 500 pptv, given that 500 pptv represented the upper limit concentration of NO_x_ in the remote troposphere. Furthermore, 500 pptv appeared to be a turning point for the external cycling to be key sources of HONO and NO_x_ (see below). The data were collected from nineteen research flights without further screening and offered a more global representativity and atmospheric variability of the external cycling and therefore supported our analysis strategy for directly exploring the external cycling of NO_x_ across high- to low-NO_x_ atmospheres. In our previous publications, we exploited data from a limited number of research flights in forested areas (i.e., RF4-5, 11, 17-18) and clean marine boundary layers (i.e., RF14, 16) and discussed the budget of HONO and specific mechanism of the external cycling^4,32^. Several studies of this kind based on aircraft observations had generally employed the budget analysis methodology for HONO to conduct case studies in clean marine air or fire plumes^21,34,38^. The reaction rate constant of the specific external cycling route implied from the missing HONO source among these reports deviated by more than two orders of magnitude, reaching no consensus in the dominant external cycling route or atmospheric variability in the external cycling^4,21,32,34,38^.

**Fig. 2:**
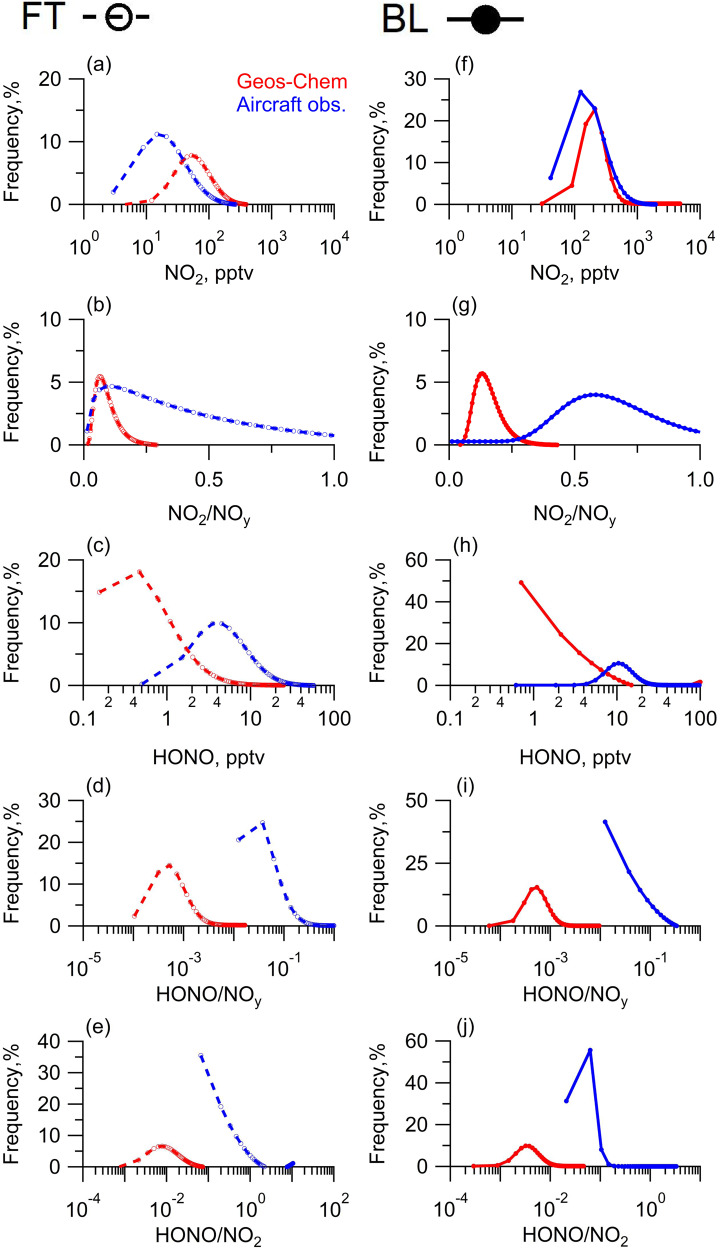
Frequency distribution of concentrations and concentration ratios of the reactive nitrogen species as measured onboard the C-130 research aircraft and simulated by GEOS-Chem. **a**–**e** Distribution of NO_2_ concentration, the concentration ratio of NO_2_/NO_y_, HONO concentration, the concentration ratio of HONO/NO_y_, and the concentration ratio of HONO/NO_2_ in the free troposphere (FT), respectively. **f**–**j** Distribution of NO_2_ concentration, the concentration ratio of NO_2_/NO_y_, HONO concentration, the concentration ratio of HONO/NO_y_, and the concentration ratio of HONO/NO_2_ in the boundary layer (BL), respectively. The red lines with circles represent the GEOS-Chem model predictions. The blue lines with circles represent our aircraft observations. Open circles and solid circles represent data points from the FT and the BL, respectively. Dashed lines and solid lines connect adjacent data points.

The NO_2_ mixing ratios ranged from 10 pptv to 14.2 ppbv, with median values of approximately 30 pptv in the FT and 218 pptv in the BL (Fig. 2 and Table S2). While ppbv levels of NO_2_ were occasionally found as we flew through urban and industrial plumes, the median values of both HONO and NO_2_ were not affected by these high values and were still representative of the background troposphere. The median NO_2_ in the BL and the FT agreed with the established NO_2_ distribution in these background atmospheres^5,21,26,38,40–42^. The median HONO mixing ratio was approximately 7.0 pptv in the FT background and 12.1 pptv in the BL background (Fig. 2 and Table S2). Our data provided the first illustrations of the HONO distribution in such a variety of low-NO_x_ atmospheres. The median HONO in the BL was among the lowest ever reported in specific low-NO_x_ environments, such as in snow/ice-covered polar areas^20,27–29^, pristine terrestrial boundary layers^32,43^, and clean marine boundary layers^4,23,25,38^. The median HONO in the FT was slightly lower than values reported from other aircraft observations in low-NO_x_ atmospheres^41,43^ but was one or two orders of magnitude lower than those observed in plumes^34,44^.

The GEOS-Chem model was used to provide a benchmark for the concentration ratios among reactive nitrogen species, such as the HONO/NO_y_ (≡NO_x_ + NO_z_) ratio, NO_2_/NO_y_ ratio and HONO/NO_2_ ratio. The emission inventory applied in GEOS-Chem overestimated NO_x_ emissions^45^. However, this drawback might not interfere with the model simulation of the HONO/NO_2_ ratio and NO_2_/NO_y_ ratio in various low-NO_x_ air masses since the reactive nitrogen species aged to reach their photosteady state (PSS) in the low-NO_x_ atmosphere. Even with the anticipated model overestimation of NO_2_ and consequently overestimation of NO_z_ (Fig. S2), the HONO concentration, HONO/NO_y_ ratio and NO_2_/NO_y_ ratio were substantially underestimated in the model (Fig. 2), indicating the external cycling of NO_x_. Moreover, model underestimation of the HONO/NO_2_ ratio and HONO/NO_y_ further revealed that HONO might be an intermediate in the external cycling of NO_x_, as HONO regenerated in the external cycling would rapidly photolyze to produce NO_x_ (Fig. 2). It was speculated that the relatively slow turnover rate of NO_x_ to produce HONO was associated with a low HONO/NO_2_ ratio (<0.05, as commonly observed in high-NO_x_ atmospheres^9^) to balance the rapid turnover rate of HONO to produce NO_x_ via HONO photolysis and the Leighton cycle. Herein, a much higher HONO/NO_2_ ratio relative to the PSS prediction in GEOS-Chem indicated a net turnover of HONO to produce NO_x_ in the external cycling and presented HONO as an intermediate product. Nevertheless, direct regeneration of NO_x_ in the external cycling bypassing HONO intermediate could not be excluded.

The external cycling route of NO_x_ with NO_z_ as a precursor and HONO as an intermediate could be proposed based on the synthesized evidence from the concentration ratios of reactive nitrogen species. Anthropogenic emission perturbation, internal cycling mechanisms, and measurement interferences to reconcile the model–observation discrepancies could be safely excluded. First, anthropogenic emissions of HONO and NO_x_ perturbed the distribution of HONO within a transport height of approximately 300 m while perturbing the distribution of NO_2_ within the boundary layer^16,19,32^. The nineteen research flights spent over 85% of the time 600 m above the ground surface in the BL and over 45% of the time in the FT. Hence, anthropogenic emissions of HONO and NO_x_ could be excluded as the major reason for the model–observation discrepancies in the concentration ratio of reactive nitrogen species. Second, the photosensitization reactions of NO_2_ produced HONO, with photo-enhanced rates several-fold higher at noon in low-NO_x_ conditions than at nighttime in high-NO_x_ conditions;^7,8,10^ however, they were still too slow to account for the high HONO/NO_2_ ratio observed (Fig. 2). In fact, fully reconciling model–observation discrepancies in the HONO/NO_2_ ratio required a reactive uptake coefficient of NO_2_ to produce HONO to be in the order of magnitude of 10^-3^, which was nearly two orders of magnitude higher than the proposed photo–enhanced values at noon in low-NO_x_ conditions^7,10^. In addition, such photosensitization reactions were not related to the model–observation discrepancy in the NO_2_/NO_y_ ratio. Finally, a substantially positive measurement interference in HONO coupled with negative measurement interferences in NO_z_ species, such as HNO_3_ and pNO_3_, might improve the model–observation agreement for both the HONO/NO_2_ ratio and NO_2_/NO_y_ ratio. However, such species-dependent interference was not practical, as any positive (or negative) nitrite anion interference in HONO measurement would also result in a positive (or negative) interference in pNO_3_ and HNO_3_ measurements. A comparison of our LPAP HONO measurements with those by the DOAS instrument showed reasonable consistency in the concentration range beyond the detection limit of both instruments, but DOAS showed a smaller value relative to LPAP in the lower concentration range^4^. Potential chemical interferences for LPAP HONO, including HNO_4_ and particulate nitrite, were not directly measured. PSS HNO_4_ interference calculations suggested that it only caused minor interference (<15% of signal)^32^. The low partitioning ratio of particulate nitrite over HONO and low sampling efficiency of particulate nitrite in the LPAP system also suggested minor interferences (<1% of signal)^46–48^. Therefore, our HONO measurement had only been corrected for PSS HNO_4_ interference and therefore, the LPAP HONO measurement appeared to be reasonably reliable in our campaign. Measurements of pNO_3_ and HNO_3_ had not been corrected for PSS HNO_4_ interference since the interference was small relative to the signals. Similarly, reliable NO_x_ measurements have been widely reported in the literature^40^. Although potential positive interference was not totally excluded^49^, model underestimation of such as the HONO/NO_2_ ratio would be even worse assuming potential positive interference of NO_2_. As such, although measurement interferences could not be completely excluded for HONO, NO_x_, pNO_3,_ and HNO_3_ measurements, the potential interferences were either very small or did not show the species-dependent characteristics required to reconcile the model–observation discrepancies. These analyses further supported the external cycling as a proper cause to reconcile the model–observation discrepancies.

Helas and Warneck and Ye et al. deduced that the lack of expected daytime minima of NO_2_ and HONO might be additional critical evidence for the external cycling, at least for the low-NO_x_ marine boundary layer^4,26^. Bell-shaped diurnals of HONO and NO_2_ were observed as critical evidence for the external cycling powered by snow photochemistry in polar areas^29,50^. Their deduction followed a budget analysis of NO_2_ and HONO. The oxidation of NO_2_ was a major sink for NO_2_, with the rate scaling with the radical concentration or photolysis frequency of O_3_ (jO^1^D). Additionally, partitioning of NO_2_ over NO was also promoted by fast photolysis of NO_2_ to produce NO at noon, as determined in the Leighton cycle^40,42^. Both chemical processes contributed to a noontime minimum of NO_2_. For HONO, daytime photolysis was the largest budget term and dominated the diurnal profile^1,2^. Therefore, typical U-shaped diurnal profiles for NO_2_ and HONO were expected and generally observed in high-NO_x_ atmospheres^1,2,40,42^. External cycling of NO_2_ and HONO might compensate for their daytime losses and result in atypical diurnal profiles of HONO and NO_2_, including flat and even bell-shaped profiles depending on the rate of external cycling^4,22,25^.

**Fig. 3:**
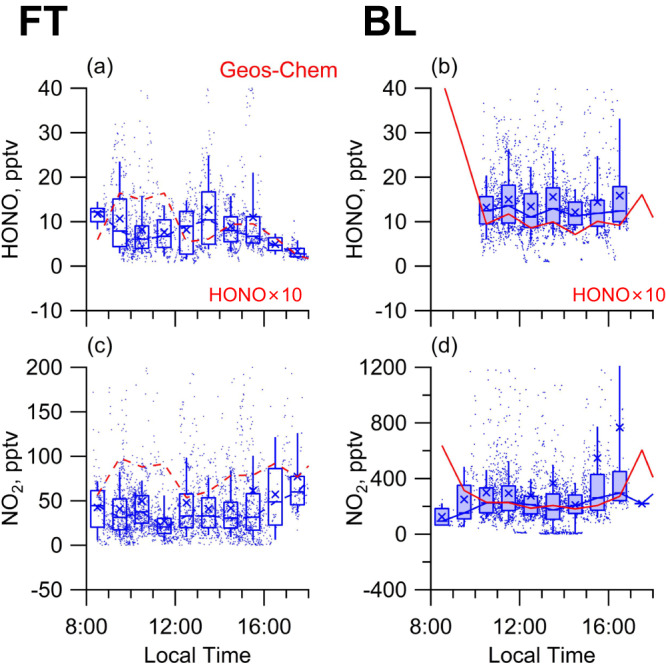
Diurnal profiles of HONO and NO_2_ as measured onboard the C-130 research aircraft and simulated by GEOS-Chem. **a**, **b** Diurnal profiles of HONO in the free troposphere (FT) and boundary layer (BL), respectively. **c**, **d** Diurnal profile of NO_2_ in the FT and BL, respectively. The red dashed and solid lines represent the median values of the GEOS-Chem model predictions. The blue boxes represent, from top to bottom, the 75th, 50th, and 25th percentiles; the whiskers above and below the boxes represent the 90th and 10th percentiles; and the cross represents the mean value of our aircraft observations. Open boxes and solid boxes represent data points from the FT and the BL, respectively. The blue dashed lines and solid lines connect adjacent median values.

Although the 19 research flights sampled various airmasses over a large geographic area across a variety of chemical regimes in the atmosphere during different hours of the day, typical photochemical peaks in radicals and jO^1^D were still observed (Fig. S3), and therefore the typical diurnals of HONO and NO_2_ were expected. However, we observed atypical diurnal profiles that lacked daytime minima of NO_2_ and HONO in more general BL, adding to the previous observation of the same kind in the clean marine boundary layer and polar boundary layer and first in the FT (Fig. 3). This observation also differed from the GEOS-Chem simulation which excluded external cycling and simulated the expected diurnal profiles of NO_2_ and HONO, especially in the BL (Fig. 3). Therefore, the atypical diurnal profiles of NO_2_ and HONO provided further observational evidence of the temporal distribution of reactive nitrogen to verify the external cycling of NO_x_ and HONO. Notably, neither of the internal cycling mechanisms, such as the photosensitization reaction of NO_2_, nor potential measurement interferences of HONO or NO_2_, were able to reconcile model–observation discrepancies in the diurnal profiles of NO_2_ and HONO.

### Chemical model evaluation of external cycling

As the GEOS-Chem did not compile detailed radical chemistry along the oxidation mechanism of VOCs^51,52^, a nearly explicit chemical model (MCM version 3.3.1, http://mcm.leeds.ac.uk/MCM/) was adapted to simulate the photochemical evolution of a random power plant plume (Fort Martin station power plant plume captured in RF10, Methods) to represent nitrogen cycling photochemistry across various NO_x_ regimes over a large geographic area. The objectives of the chemical model simulation were to explore the fundamental characteristics of external cycling (i.e., HONO being an intermediate, determinative role of external cycling in the observed high ratio of HONO/NO_2_ and NO_2_/NO_y_, and the perturbation of external cycling on OH photochemistry) along with the aging of the plume. To be more specific, measurements in the power plant plume were chosen to initialize our model, while chemical conditions in the low-NO_x_ atmosphere were carefully summarized to constrain the model. To focus on the chemical evolution of composition in the plume, a conceptual photochemical evolution under solar noon conditions was simulated in our model. To avoid discussion on any specific external cycling mechanism and related arguments on the reaction rate of specific external cycling routes, a proxy mechanism for the external cycling employing pNO_3_ as a representative of NO_z_ species and the precursor of HONO and NO_x_ in the external cycling was included in the chemical model scheme. The pNO_3_ photolysis rate or the reaction rate of a general NO_z_ species was set up based on the assumption that the external cycling could fully account for the unknown source of HONO or NO_2_. Previous field observations or laboratory measurements of the photolysis rate constant were not referred to or compared with, as only a proxy mechanism, rather than a specific mechanism, of the external cycling was the very core of the discussion. Three independent models were run: one excluded the external cycling of NO_x_ and HONO (model S0), and the other two included external cycling, with/without HONO as a NO_x_ intermediate (model S1-S2). Since the external cycling was verified and HONO was identified as an intermediate product based on our observations, chemical model S1 was expected to best represent our observations on the distribution of HONO and NO_2_ in varied NO_x_ regimes.

**Fig. 4:**
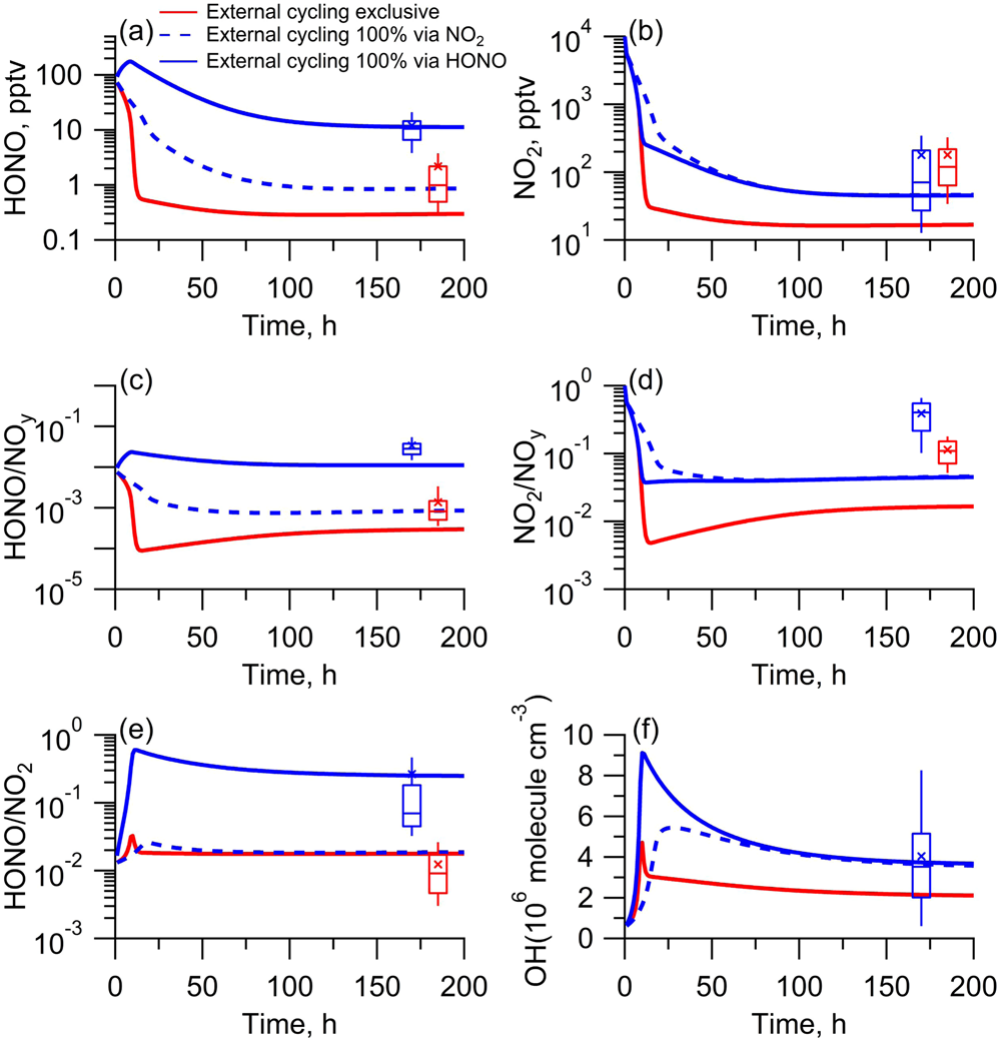
Distribution patterns of concentration and concentration ratios of reactive nitrogen species, and OH radicals during plume aging as simulated by the MCM model. **a**, **b** Concentrations of HONO and NO_2_, respectively. **c**–**e** Concentration ratio of HONO/NO_y_, NO_2_/NO_y_, and HONO/NO_2_, respectively. **f** Concentration of OH radicals. The red line represents the model S0, which excludes external cycling. The blue line and blue dashed line present the model S1 and S2, which include the proxy mechanism for external cycling with HONO yields of 100% (0% yield for NO_2_) and 0% (100% yield for NO_2_), respectively. The blue and red boxes represent the results of our aircraft observations and GEOS-Chem simulations, respectively. From top to bottom of the box, the 75th, 50th, and 25th percentiles are shown; the whiskers above and below the boxes represent the 90th and 10th percentiles; and the cross represents the mean values.

The kinetic curve of reactive nitrogen species during the aging of the Fort Martin station power plant plume was shown in Fig. 4. NO_2_ went through quasi-exponential decay in the initial period and then approached a steady period with a stabilized NO_2_/NO_y_ ratio and any other concentration ratios of reactive nitrogen species (Fig. 4). The initial period simulated reactive nitrogen photochemistry in a fresh plume or high-NO_x_ atmosphere where the oxidation of NO_x_ to form NO_z_ species dominated the NO_x_ budget, leading to rapid NO_2_ decay. The steady period mimicked reactive nitrogen photochemistry in aged plumes or in low-NO_x_ atmospheres where the oxidation loss and regeneration of NO_2_ from the external cycling approached an equilibrium state, resulting in relatively consistent NO_2_ levels that had been persistently observed in various low-NO_x_ atmospheres in our study and in the literature^4,21,40^.

The counterbalancing role of NO_x_ regeneration in the external cycling extended the NO_x_ lifetime and sustained comparable NO_x_ abundance in aged airmasses or remote atmospheres. It also challenged the current chemical model scheme involving the continuous oxidative decay of NO_x_ and a small NO_x_ regeneration rate, leading to extremely low NO_x_ abundance in the tropospheric background. In model S0, the NO_2_/NO_y_ ratio was not negligible but was extremely low (Fig. 4) due to the low regeneration rate of NO_x_ via transport and the thermal decomposition of PAN, even with higher PAN and higher rates of PAN decomposition applied in the model than those obtained from our aircraft observations (Fig. S4). The external cycling of NO_x_ in model S1 or S2 better agreed with the observed NO_2_ concentrations and concentration ratios of NO_2_/NO_y_ in low-NO_x_ environments, confirming the determinative role of the external cycling in the distribution of NO_2_/NO_y_ ratio especially in low-NO_x_ atmospheres. Model S1 also better agreed with the HONO concentration, HONO/NO_y_ ratio, and HONO/NO_2_ ratio, confirming HONO as an intermediate in the external cycling. Compared to other NO_x_ regeneration mechanisms, such as thermal decomposition and photolysis of PAN (gaseous HNO_4_, organic nitrates, and nitric acid), the external cycling involving HONO as an intermediate proceeded far more rapidly, especially in low-NO_x_ atmospheres.

**Fig. 5:**
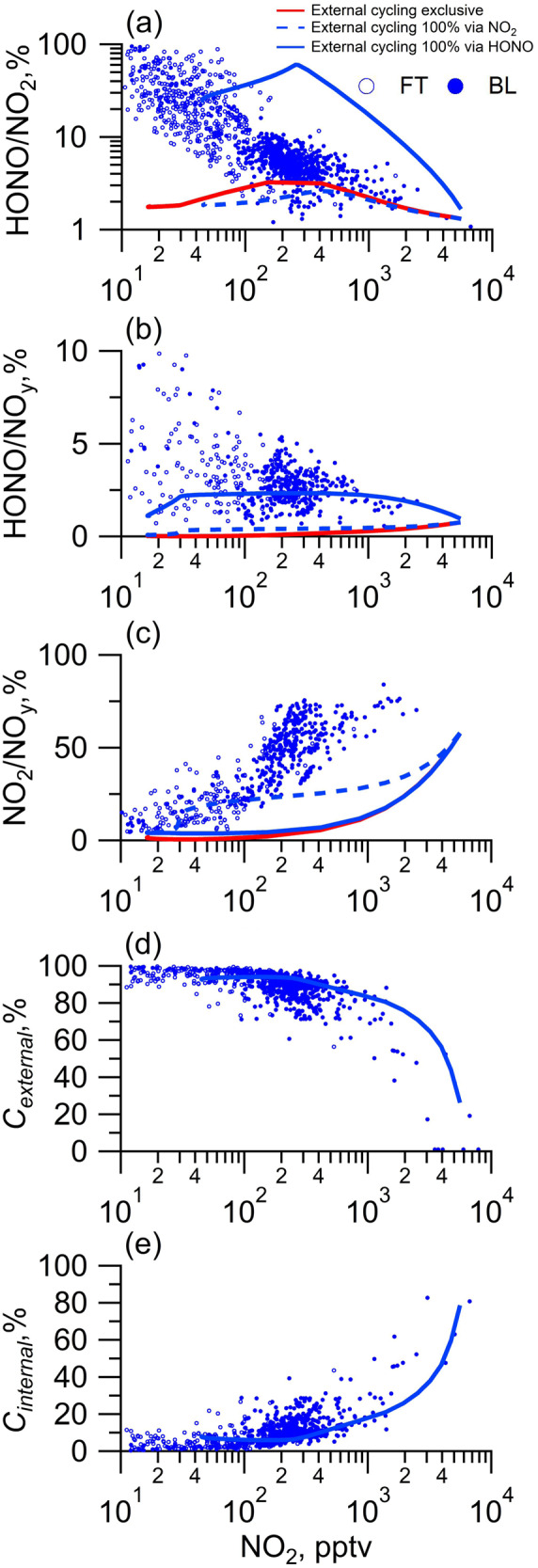
Scatter plots of the concentration ratios of reactive nitrogen species, and contribution of external and internal cycling, against NO_2,_ as measured onboard the C-130 research aircraft and simulated by the MCM model. **a**–**c** Concentration ratios of HONO/NO_2_, HONO/NO_y_, NO_2_/NO_y_, respectively. **d**, **e** External source contribution (*C*_*external*_) and internal source contribution (*C*_*internal*_), respectively. Open circles and solid circles represent measured data points from the free troposphere (FT) and the boundary layer (BL), respectively. The red line represents model S0, which excludes external cycling. The blue line and blue dashed line present the model S1 and S2, which include the proxy mechanism for external cycling with HONO yields of 100% (0% yield for NO_2_) and 0% (100% yield for NO_2_), respectively.

To note, our observation and modeling consistently demonstrated the environmental variability pattern in the role of external cycling as the plume aged, and our modeling with the external cycling better captured the observed ratios of HONO/NO_2_, HONO/NO_y_, NO_2_/NO_y_ as NO_x_ decreased (Fig. 5). These environmental variability patterns were mainly rationalized by the accumulation of NO_z_ (the external cycling precursor) as the plume aged. Consequently, an increasing trend in the HONO/NO_2_, HONO/NO_y_ ratio and therefore a relatively strong external cycling contribution (*C*_*external*_) to the budget of HONO and NO_x_ as NO_x_ decreased were observed (see Calculation of *C*_*internal*_ and *C*_*external*_ in Method section). The environmental variability of the rate constant of the external cycling, i.e., the high rate constant of the external cycling in low NO_x_ atmospheres, might also contribute to the environmental variability patterns^30^. Although the environmental variability in the rate constant of the external cycling was not considered in our box model scheme, our model reasonably captured the observations across NO_x_ regimes. Therefore, both our observations and the model simulation illustrated increasing perturbations on atmospheric budgets of reactive nitrogen species by the external cycling as NO_x_ decreased.

Frankly speaking, it is difficult to comprehend the observed trend of *C*_*external*_ as NO_x_ decreases since it goes against the traditional theories in which internal cycling dominates the HONO budget. The shifting of the HONO/NO_2_ ratio as NO_x_ decreased in the high-NO_x_ atmosphere was so slow and limited in a narrow range, typically from 0.01 to 0.1 (Figs. 4, 5), that it might have been considered unchanged given the observational uncertainties. The relationship between HONO and NO_x_ was strengthened by numerous observations in high-NO_x_ atmospheres^1,9,13^, and the dominant role of the internal cycling tended to be mistakenly extrapolated to low-NO_x_ chemical regimes as a consequence. Our characterization of the chemical-regime-dependent contribution of the external cycling better summarized the environmental variability feature of the external cycling and properly addressed the observational argument regarding missing HONO sources in high-NO_x_ atmospheres and low-NO_x_ atmospheres^44^.

The increasing contribution of the external cycling to the budget of HONO and NO_x_ as NO_x_ decreased might have amplified its perturbation on OH photochemistry because OH production was mostly sensitive to the abundance shifting of NO_x_ in the low-NO_x_ atmosphere. In addition, the daytime compensation of NO_x_ by the external cycling might have further amplified its perturbation role because the photochemical production of OH overlapped with the external cycling of NO_x_ and HONO during the daytime. This point was illustrated in the model underestimation of OH. Due to model S0 omission of the external cycling, the model underestimated OH, and this underestimation was more apparent in a low-NO_x_ atmosphere than in a high-NO_x_ atmosphere (Fig. 4). The model underestimation of OH was also confirmed to be more closely linked with primary OH production via photolysis of HONO (driven mainly by the internal cycling) in a high-NO_x_ atmosphere but more closely associated with NO_x_-catalyzed secondary OH production driven by the external cycling of NO_x_ in a low-NO_x_ atmosphere. The S0 model underestimation of OH abundance reached approximately 41% in a low-NO_x_ atmosphere (Fig. 4), comparable to a previous CTM evaluation, which showed a perturbation of the NO_x_ budget and therefore the OH budget by approximately 40% via a proxy cycling mechanism in the marine boundary layer^35^.

Overall, the results of our analysis indicate the dominant role of external cycling in the chemical budget of reactive nitrogen in low-NO_x_ atmospheres and its significant impact on oxidant photochemistry, thus calling for an urgent and significant revision in our understanding of the atmospheric chemistry of reactive nitrogen and oxidants. To advance this goal, extensive research efforts are needed, for example, laboratory work to better parameterize the kinetics and mechanisms of dominant external cycling routes, including but not limited to pNO_3_ photolysis, and field measurements to establish spatial and temporal distributions of reactive nitrogen species that are only sporadically available at this time, especially for low-NO_x_ atmospheres^5,17,34,53,54^.

## Methods

### Aircraft observation

A total of 19 research flights were conducted, mostly from 8:00 to 18:00 local time, in various low-NO_x_ troposphere environments. The raw data from the first 15 min after taking off and the last 15 min before landing were excluded from the analysis to avoid interference from pollution at the airport and were then averaged for 3 min for further analysis.

O_3_ and NO_x_ were measured using chemiluminescence methods^55,56^. HONO, HNO_3_, and pNO_3_ were measured using a wet-chemistry method similar to LOPAP^57^. Aerosol number-size distributions were measured using a scanning mobility particle sizer (SMPS) and an ultrahigh sensitivity aerosol spectrometer (UHSAS)^58^. Alkyl nitrates, PANs, and VOCs were measured by a trace organic gas analyzer (TOGA)^59^. Free radicals were measured using a selected-ion chemical-ionization mass spectrometer (SICIMS)^60^. Photolysis frequencies were calculated from measurements of a scanning actinic flux spectroradiometer (SAFS)^61^.

### Calculation of *C*_*internal*_ and *C*_*external*_

The internal cycling included heterogeneous reactions of NO_2_ on ambient surfaces, gas-phase reaction between NO and OH radicals, HONO photolysis, and gas-phase reaction between HONO and OH radicals. The chemical reactions are listed as follows.

$${{{{{{\rm{NO}}}}}}}_{2}+{{{{{\rm{surface}}}}}}\to {{{{{\rm{HONO}}}}}}+{{{{{\rm{other\; products}}}}}}$$

$${{{{{\rm{HONO}}}}}}+{hv} \, ({{{{{\rm{\lambda }}}}}} \, < \, 400{{{{{\rm{nm}}}}}})\leftrightarrow {{{{{\rm{OH}}}}}}+{{{{{\rm{NO}}}}}}$$

$${{{{{\rm{HONO}}}}}}+{{{{{\rm{OH}}}}}}\to {{{{{{\rm{H}}}}}}}_{2}{{{{{\rm{O}}}}}}+{{{{{{\rm{NO}}}}}}}_{2}$$

The internal source of HONO (${P}_{{internal}}$) is calculated with the equation below:

1 $${P}_{{internal}}={k}_{R1}\left[{{{{{{\rm{NO}}}}}}}_{2}\right]+{k}_{R2}\left[{{{{{\rm{OH}}}}}}\right]\left[{{{{{\rm{NO}}}}}}\right]$$

where [X] represents the concentration of species X in molecule cm^−3^; ${k}_{R2}$ is the rate constant of NO with OH radicals; ${k}_{R1}$ is defined as the heterogeneous uptake rate constant of NO_2_ on the aerosol surface and is calculated with Eq. (2).

2 $${k}_{R2}=\frac{1}{4}\times s/v\times c\times \gamma$$

where *s/v* represents the aerosol surface density, which is calculated from the particle number-size distribution by the SMPS. *c* represents the average molecular speed of NO_2_. $\gamma$ represents the uptake coefficient of NO_2_ on an aerosol particle surface. An upper limit of 10^−4^ was adopted to estimate the upper limit of internal sources of HONO^62^.

With a photolytic lifetime of approximately 10 min, the photosteady-state assumption is applicable for HONO. The external source of HONO (${P}_{{external}}$) is calculated according to the photosteady-state budget as follows.

3 $${P}_{{internal}}+{P}_{{external}}=({j}_{{HONO}}+{k}_{R3}\left[{{{{{\rm{OH}}}}}}\right])\times \left[{{{{{\rm{HONO}}}}}}\right]$$

where [X] represents the concentration of species X in molecule cm^−3^, ${j}_{{HONO}}$ represents the photolysis frequency of HONO, and ${k}_{R3}$ represents the rate constant of HONO with OH radicals. Soil and anthropogenic emissions of HONO are not included in the budget. The aircraft measurements were mostly taken ≥ 600 m above the ground in the boundary layer and up to 8000 m in the free troposphere. The air masses encountered were therefore decoupled from ground surface HONO sources such as soil emissions and heterogeneous reactions of NO_2_ at ground surfaces^63^. The high HONO/NO_2_ ratio observed here also excluded influences from primary HONO emissions except for occasional influences from power plant plumes or city plumes. Therefore, only in situ chemical reactions were considered for the internal and external cycling rate calculation in this study.

The relative contribution of the internal source and external source to the total HONO source, *C*_*internal*_ and *C*_*external*_, can be defined as follows:

4 $${C}_{{internal}}=\frac{{P}_{{internal}}}{{P}_{{internal}}+{P}_{{external}}}$$

5 $${C}_{{external}}=\frac{{P}_{{external}}}{{P}_{{internal}}+{P}_{{external}}}$$

### Nearly explicit chemical model simulation

The master chemical mechanism (MCM v3.3.1) is adapted here to simulate nitrogen cycling photochemistry and its impacts on the oxidative capacity of the atmosphere. The specific objective of the model run was to test whether the external cycling proxy mechanism, i.e., particulate nitrate photolysis or reaction of a general NO_z_ species, can reproduce the unique distribution patterns of OH, HONO, and NO_2_ in the low-NO_x_ troposphere.

The chemical scheme extracted from the MCM website was employed. The initial mechanism revisions of the MCM model include the addition of the heterogeneous production of HONO from NO_2_ reactions, dry deposition-driven removal of model-calculated OVOC intermediates and reactive nitrogen species, and a transport source for HNO_3_ and PAN to compensate for dry deposition losses of NO_y_ species^22,25^. The conversion rate of HONO from NO_2_ reactions is set to be 1.4 × 10^−5 ^s^−1^ ( ~ 0.05 h^−1^). The dry deposition-driven removal of model-calculated OVOC intermediates and reactive nitrogen species are set to be 9.0 × 10^−6 ^s^−1^ and 1.0 × 10^−5 ^s^−1^, respectively. Both the transport source rate for HNO_3_ and PAN are 18 pptv h^−1^. This model mechanism setup is referred to as the baseline model, S0, which excludes the external cycling proxy mechanism. In further revised models (model S1 and S2), we added HONO production from the proxy mechanism of the external cycling (Table S3). The production rate of HONO or NO_2_ (${P}_{{{pNO}}_{3}}$) was expressed as follows:

6 $${P}_{{{pNO}}_{3}}={[{{{{{{\rm{pNO}}}}}}}_{3}]\times j}_{{HNO}3}\times {EF}$$

where$\,{j}_{{HNO}3}$ is the photolysis frequency of HNO_3_ in its gaseous form, and the enhancement factor, *EF*, is the ratio of the photolysis rate constant of particulate nitrate relative to ${j}_{{HNO}3}$. A median *EF* of 150 and a pNO_3_/tHNO_3_ partitioning ratio of 0.5 were employed in the model to represent the average conditions for the background troposphere^30^. A HONO yield of 100% (S1, 0% yield for NO_2_) or 0% (S2, 100% yield for NO_2_) was adapted in models S1 and S2, respectively.

$$	{{{\rm{proxy}}}}\; {{{\rm{mechanism}}}}\; {{{\rm{in}}}}\; {{{\rm{Model}}}}\; {{{\rm{S}}}}1: \qquad {{{{\rm{pNO}}}}}_{3}+{hv}\mathop{\longrightarrow }\limits^{150\times {j}_{{HNO}3}}{{{\rm{HONO}}}}\\ 	 \qquad\qquad \qquad \mathop{\longrightarrow }\limits^{{j}_{{HONO}}}{{{{\rm{NO}}}}}_{2}\\ 	{{{\rm{proxy}}}}\; {{{\rm{mechanism}}}}\; {{{\rm{in}}}}\; {{{\rm{Model}}}}\; {{{\rm{S}}}}2:\qquad{{{{\rm{pNO}}}}}_{3}+{hv}\mathop{\longrightarrow }\limits^{150\times {j}_{{HNO}3}}{{{{\rm{NO}}}}}_{2}$$

To mimic the photochemical evolution of reactive nitrogen species in the typical tropospheric background and represent external cycling and its perturbation on OH photochemistry across various NO_x_ regimes over a large geographic area, the model was initialized with a NO_x_ mixing ratio of 10 ppbv, as observed in the Fort Martin station power plant plume. Concentrations of O_3_ and OH were initialized at 30 ppbv and 1.7 × 10^6 ^cm^−3^, respectively, which were typically observed in our daytime measurements. To best represent the radical chemistry for the background troposphere, an OH reactivity of approximately 5 s^−1^ was used for the model based on our aircraft measurements of VOCs and other species that consume OH, such as O_3_, NO_x_, and CO. CO, CH_4_, HCHO, and CH_3_CHO were found to be the top four contributors to OH reactivity in the background troposphere. To simplify the model, all measured VOCs, except HCHO and CH_3_CHO, were grouped with CO to obtain an equivalent OH reactivity. The oxidative mechanism of these grouped VOCs was thus not included in our model. The photosteady state was established quickly in the model under these initial conditions. Because our focus was on the photochemical cycling of HONO and NO_2_, the models were run without day-night shifts and had constant photolysis frequencies under zero solar zenith angle conditions (Table S4) to mimic the continuous 400 hr photochemical aging of the power plant plume in the background troposphere. After approximately 100 hours, the model simulation reached a stable stage. Thus, only results for the first 200 hours are shown. The model settings and initialization conditions are summarized in Tables S3 and S4.

Our model generally reproduces the observation of reactive nitrogen in various atmospheric chemical regimes. The slightly lower modeling of the HONO/NO_y_ ratio and NO_2_/NO_y_ ratio might be partly accounted for by measurement underestimations in NO_y_ species (Figs. 5 and S4). Air bubbles can deactivate the Cd columns in LPAPs, leading to potentially lower nitrate-to-nitrite conversion efficiencies; measurement underestimations in pNO_3_ and HNO_3_ from this phenomenon were recently estimated to reach twofold^23^. Incomplete NO_y_ measurements in the aircraft platform, such as measurements of other alkyl nitrates, and uncertainties in the organic nitrogen formation mechanism might be other reasons^17^. An assumed extreme case of 100% HONO yield in model S1 might be another reason, as the direct yield of NO_2_ cannot be excluded^4,25^.

### GEOS-Chem simulation

The GEOS-Chem model (version 9-02; www.geos-chem.org) regional simulations were conducted in a one-way nested grid formulation with the native GEOS-5 Forward Processing horizontal resolution of 0.25° × 0.3125° over the North America domain (130–60° W, 10–60° N). The initial and boundary conditions were obtained through a global GEOS-Chem model simulation with a coarser resolution of 2° × 2.5° (reduced from the native GEOS-5 forward processing grid). The GEOS-Chem meteorological fields were obtained from the assimilated products of the NASA Global Modeling and Assimilation Office Goddard Earth Observing System (http://gmao.gsfc.nasa.gov/products/). The model simulations had 47 vertical layers in the atmosphere. We used a nonlocal planetary boundary layer mixing scheme developed by Holtslag and Boville (1993) and implemented in GEOS-Chem by Lin and McElroy (2010)^64,65^. The tropospheric chemistry mechanism was described in Parrella et al. (2012)^66^. No further updates, including external cycling, were made. Emissions of atmospheric components included anthropogenic emissions from the Emissions Database for Global Atmospheric Research (https://edgar.jrc.ec.europa.eu), biogenic emissions of volatile organic carbons from the Model of Emissions of Gases and Aerosols from Nature inventory^67^, fire emissions from the Global Fire Emissions Database^68^, soil NO_x_ emissions^69^, and lightning NO_x_ sources^70^. The dry deposition scheme used a resistance in-series model based on Wesely (1989)^71^. The wet deposition scheme included the scavenging of soluble tracers in convective updrafts as well as the rainout and washout of soluble tracers^72^. Simulation results corresponding to the research flight track during the same period were extracted for measurement comparison.

### Supplementary information

Supplementary Information

Peer Review File

## Data Availability

The field data used in this study have been deposited and are freely available in the SAS project data archive (http://data.eol.ucar.edu/master_list/?project=SAS).

## Code availability

The full set of the MCM model mechanism can be extracted from the MCM website (https://mcm.york.ac.uk/MCM/) and the subset is also available from the corresponding author upon request.
