# Supplementary material for: Synthesizing evidence for the external cycling of NOx in high- to low-NOx atmospheres
Source: Nat Commun. 2023 Dec 2;14:7995. doi: 10.1038/s41467-023-43866-z (PMC10693570; doi:10.1038/s41467-023-43866-z)
Supplement: Supplementary file 1 — Supplementary Information [file 41467_2023_43866_MOESM1_ESM.pdf]

Supplementary information for the manuscript entitled "Synthesizing evidence for the external cycling of NO_x in high- to low-NO_x atmospheres"

Chunxiang Ye[1, *], Xianliang Zhou[2, 3], Yingjie Zhang[1,4], Youfeng Wang[1], Jianshu Wang[1], Chong Zhang[1], Robert Woodward-Massey[1,5], Christopher Cantrell[6], Roy L. Mauldin III[7-9], Teresa Campos[10], Rebecca S. Hornbrook[10], John Ortega[10], Eric C. Apel[10], Julie Haggerty[10], Samuel Hall[10], Kirk Ullmann[10], Andrew Weinheimer[10], Jochen Stutz[11], Thomas Karl[12], James N. Smith[13], Alex Guenther[13], Shaojie Song[14]

[1]State Key Joint Laboratory of Environmental Simulation and Pollution Control (SKL-ESPC), College of Environmental Sciences and Engineering, Peking University, Beijing, China.

[2]Wadsworth Center, New York State Department of Health, Albany, NY, USA

[3]Department of Environmental Health Sciences, State University of New York, Albany, NY, USA

[4]School of Ecology and Nature Conservation, Beijing Forestry University, Beijing, China

[5]Department of Chemistry, University of Leeds, Leeds, UK

[6]Université Paris-est Créteil, LISA (Laboratoire Interuniversitaire des Systèmes Atmosphériques), Paris, France

[7]Center for Atmospheric Particle Studies, Carnegie Mellon University, Pittsburgh, PA, USA

[8]Department of Chemistry, Carnegie Mellon University, Pittsburgh, PA, USA

[9]Department of Atmospheric and Oceanic Sciences, University of Colorado Boulder, Boulder, CO, USA

[10]National Center for Atmospheric Research, Boulder, CO, USA

[11]Department of Atmospheric and Oceanic Sciences, University of California, Los Angeles, CA, USA

[12]Institute for Meteorology and Geophysics, University of Innsbruck, Innsbruck, Austria

[13]Earth System Science, University of California, Irvine, CA, USA

[14] State Environmental Protection Key Laboratory of Urban Ambient Air Particulate Matter Pollution Prevention and Control & Tianjin Key Laboratory of Urban Transport Emission Research, College of Environmental Science and Engineering, Nankai University, Tianjin, China

*Corresponding author E-mail address: c.ye@pku.edu.cn

This file includes:

Supplementary Figures 1-4

Supplementary Tables 1-4

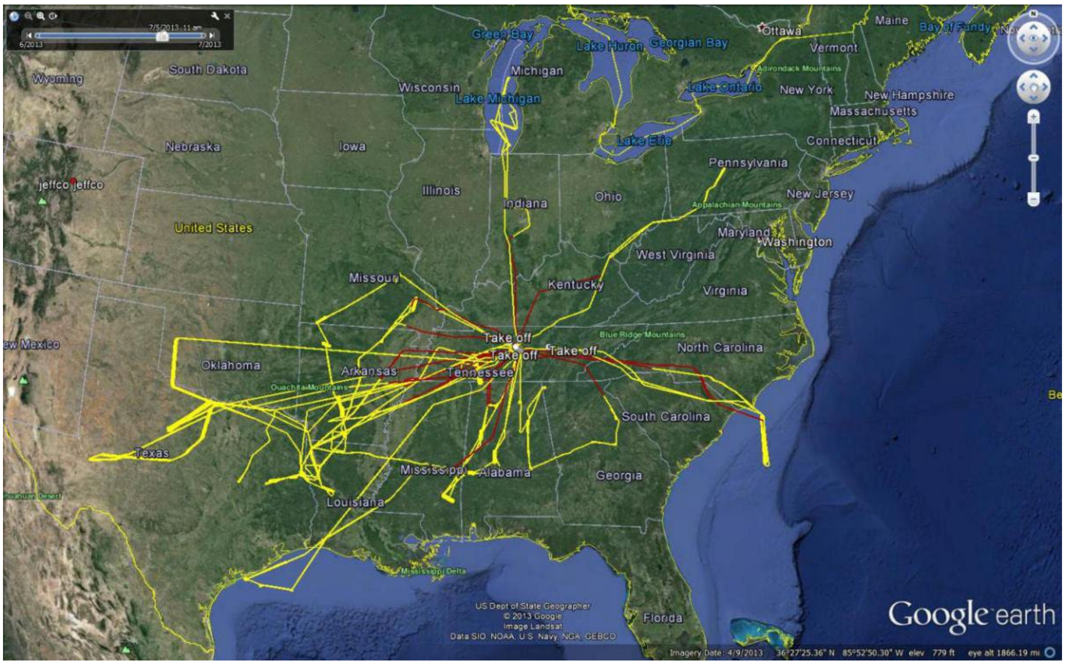

Fig. S1 | Flight track map of the NSF/NCAR C130 research aircraft from June 1 to July 15, 2013. Figure origin: https://data.eol.ucar.edu/master_lists/generated/sas/.

Map data ©2013 Google.

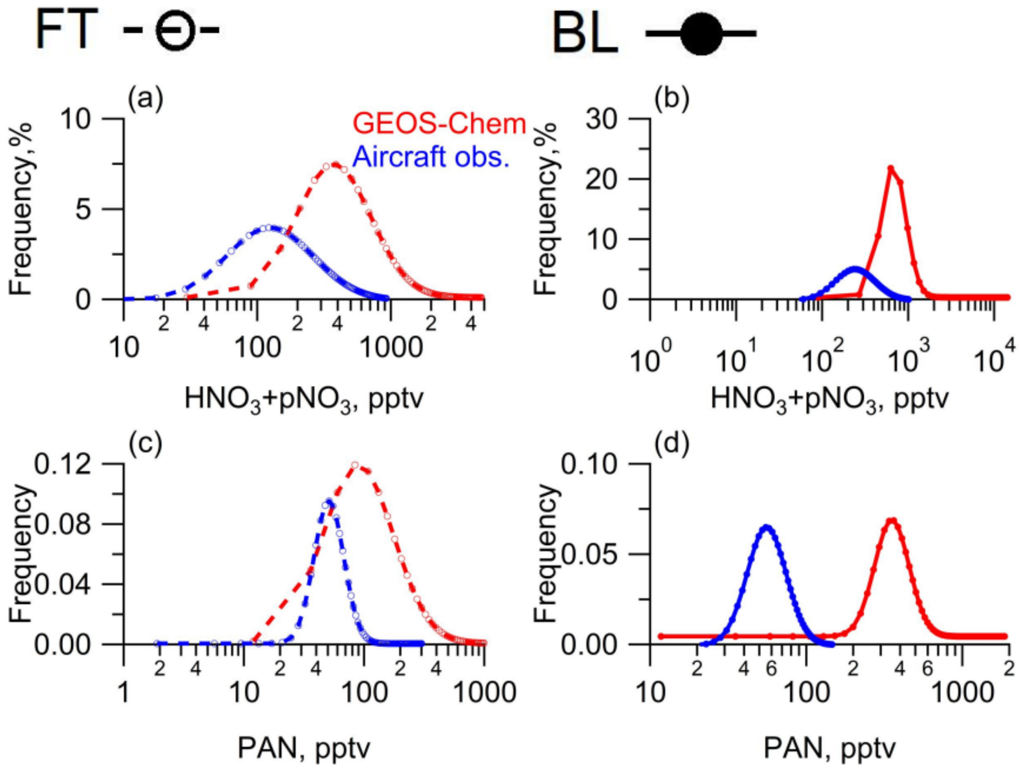

Fig. S2 | Frequency distribution of HNO_3+pNO_3 and PAN as measured on board the C-130 research aircraft and simulated by GEOS-Chem. (a) frequency distribution of HNO_3+pNO_3 in the free troposphere (FT), (b) frequency distribution of HNO_3+pNO_3 in the boundary layer (BL), (c) frequency distribution of PAN in the FT, and (d) frequency distribution of PAN in the BL. The red lines with circles represent GEOS-Chem model predictions. The blue lines with circles represent our aircraft observations. Open circles and solid circles represent data points from the FT and the BL, respectively.

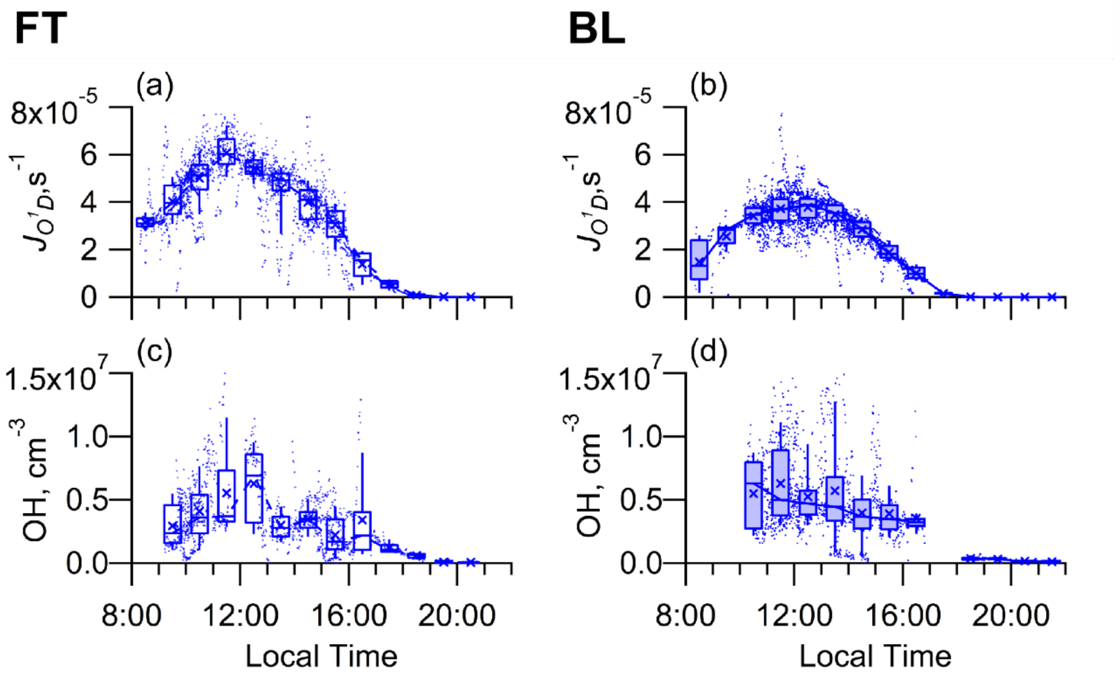

Fig. S3 | Diurnal profiles of the O_3 photolysis frequency (J_{O1D}) and OH radicals in the FT and the BL as measured on board the C-130 research aircraft. (a) diurnal profiles of J_{O1D} in the free troposphere (FT), (b) diurnal profiles of J_{O1D} in the boundary layer (BL), (c) diurnal profiles of OH radicals in the FT, and (d) diurnal profiles of OH radicals in the BL. The boxes in each panel represent, from top to bottom, the 75th, 50th, and 25th percentiles; the whiskers above and below the boxes represent the 90th and 10th percentiles; and the cross represents the mean value. Open boxes and solid boxes represent data points from the FT and the BL, respectively. The dashed lines and solid lines connect adjacent medians.

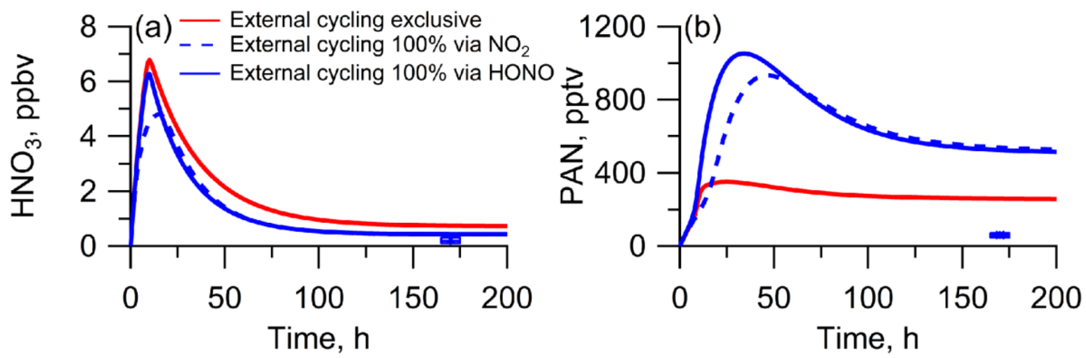

Fig. S4 | Distribution of HNO_3 (a) and PAN (b) during plume aging as simulated by the MCM model. The red line represents model S0, which excludes the external cycling proxy mechanism. The blue line and blue dashed line present the model S1 and model S2, which include the external cycling proxy mechanism with a HONO yield of 100% (0% yield for NO_2) and a HONO yield of 0% (100% yield for NO_2), respectively. The boxes represent, from top to bottom, the 75th, 50th, and 25th percentiles; the whiskers above and below the boxes represent the 90th and 10th percentiles; and the cross represents the mean value of our aircraft observations. NO_x regeneration from PAN, even when it was model-optimized, could not reconcile the model–observation discrepancy in the NO_2/NO_y ratio.

Table S1: Summary of flight missions and flight tracks.

MISSION	START TIME	END TIME	MISSION SUMMARIES
RF01	2013/6/3 14:00	2013/6/3 18:34	Smyrna to ROSE site in Central AL and then return to Smyrna.
RF02	2013/6/5 14:44	2013/6/5 21:30	Flight to Texas/Louisiana border for multiple (4) stacked racetracks in the boundary layer (BL).
RF03	2013/6/8 14:06	2013/6/8 21:05	Flight to Missouri/Arkansas for multiple (2 to 4) stacked racetracks in the BL at four forested sites.
RF04	2013/6/12 15:14	2013/6/12 22:28	Flight to racetrack sites in Alabama and Mississippi.
RF05	2013/6/14 15:05	2013/6/14 21:31	Flight #2 to Alabama/Mississippi for multiple (2 to 4) stacked racetracks in the BL at four forested sites.
RF06	2013/6/19 14:59	2013/6/19 22:08	Sampling of high altitude/dry air over Texas and East Texas power plant plumes.

RF07	2013/6/20 16:29	2013/6/20 23:38	Sample power plant and other plumes in a heavily impacted region of the Ohio River Valley.
RF08	2013/6/22 15:16	2013/6/22 22:15	Sample Birmingham and NE Texas power plant plumes.
RF09	2013/6/24 15:09	2013/6/24 22:21	Vertical profile in very dry air over Texas. Sampled possible biomass burning plume at 15 kft.
RF10	2013/6/27 15:03	2013/6/27 21:53	Sampled high elevation air over TX/OK with likely BB influence. Sampling at low elevations near Texarkana and Martin Lake power plants.
RF11	2013/6/29 15:20	2013/6/29 21:02	Sample Birmingham, Gaston power plant, and Atlanta power plant plumes.
RF12	2013/7/1 14:14	2013/7/1 21:22	Flight to Texas Gulf Coast.
RF13	2013/7/4 15:19	2013/7/4 22:09	Sampled Hg plume at high levels between Smyrna and El Dorado, Texas.

RF14	2013/7/5 15:08	2013/7/5 21:44	Sampled clean marine air over the Atlantic Ocean.
RF15	2013/7/7 15:11	2013/7/7 22:01	To study Chicago outflow over Lake Michigan in and above BL; (2) To sample Indianapolis plume in the BL; (3) To measure biogenic VOC flux over forested regions in South Indiana.
RF16	2013/7/8 15:04	2013/7/8 21:48	To sample clean marine air over the Atlantic Ocean; (2) to sample BL air masses over the forest and the land-sea transition zone.
RF17	2013/7/11 15:08	2013/7/11 21:57	Sampled aged Canadian biomass burning plume over the eastern border of the Ozarks.
RF18	2013/7/12 20:32	2013/7/13 3:37	Sampled air in multiple stacked racetracks over a forested region to establish vertical profiles during the day; (2) to study nighttime evolution of HONO in the daytime BL remnant and the free troposphere.
RF19	2013/7/14 14:05	2013/7/14 21:08	Flight to Eastern Arkansas.

Table S2: Medians and means (±SD) of the mixing ratios of HONO, NO_2, PAN, and HNO_3+pNO_3 in our aircraft observations and simulated by GEOS-Chem.

parameters	HONO, pptv<br>Median<br>Mean(±SD)	NO_2, pptv<br>Median<br>Mean(±SD)	PAN, pptv<br>Median<br>Mean(±SD)	HNO_3+pNO_3, pptv<br>Median<br>Mean(±SD)
Free troposphere	7.0	30	55	189.1
Aircraft obs.	9.2(±8.0)	41 (±87)	59 (±23)	221 (±127)
Boundary layer	12.1	219	60	319
Aircraft obs.	14.1(±8.5)	340(±764)	60 (±17)	308 (±136)
Free troposphere	1.0	80	146	566
GEOS-Chem	1.8(±2.4)	93 (±76)	194 (±169)	739(±558)
Boundary layer	1.0	218	379	757
GEOS-Chem	2.67(±8.0)	291(±401)	410(±212)	1132 (±1179)

Table S3: MCM model mechanisms in specific model run scenarios.

MCM v3.3.1	Mechanism revisions
S0, external cycling exclusive model	MCM v3.3.1 + heterogeneous NO_2 → HONO conversion ($\gamma = 1\times10^{-4}$) + dry deposition removal + transport of HNO_3 and PAN[1]
S1, external cycling 100% via HONO	S0 + pNO_3 photolysis → HONO with 100% yield (0% NO_2 yield) and EF = 150[2]
S2, external cycling 100% via NO_2	S0 + pNO_3 photolysis → HONO with 0% yield (100% NO_2 yield) and EF = 150[2]

Table S4: Initialization conditions of all MCM model runs.

Parameters/variables	Settings
NO_2 (variable)	Initialized at 10 ppb
OH (variable)	Initialized at 1.7×10^{6} cm^{-3}
O_3 (variable)	Initialized at 30 ppb
Other species concerning OH reactivity (parameters)	By constraining CO=115 ppb, CH_4=1.84 ppm, HCHO=1.88 ppb, and CH_3CHO=0.415 ppb, we initialize the model with OH reactivity of ~5 s^{-1}. Modeled OH reactivity is allowed to change with NO_x decreases.
Photolysis frequencies (parameters)	Set to cloud-free and zero solar zenith angle conditions, e.g., $j(HONO)$ =1.36×10^{-3} s^{-1}, $j(O^1D)$ =2.40×10^{-5} s^{-1}, $j(NO_2)$ =6.15×10^{-3} s^{-1}.
Transport sources (parameters)	HNO_{3i} = 18 pptv h^{-1}<br>PAN_i = 18 pptv h^{-1}
Partitioning factor ($pNO_3/tHNO_3$) (parameter)	0.5

Supplementary References

1. Ye, C.X., Heard, D.E. & Whalley, L.K. Evaluation of Novel Routes for NOx Formation in Remote Regions. *Environmental Science & Technology* **51**, 7442-7449 (2017).
2. Ye, C.X., Zhang, N., Gao, H.L. & Zhou, X.L. Photolysis of Particulate Nitrate as a Source of HONO and NOx. *Environmental Science & Technology* **51**, 6849-6856 (2017).
